# Combining multimodal adaptive optics imaging and angiography improves visualization of human eyes with cellular-level resolution

**DOI:** 10.1038/s42003-018-0190-8

**Published:** 2018-11-14

**Authors:** HaeWon Jung, Tao Liu, Jianfei Liu, Laryssa A. Huryn, Johnny Tam

**Affiliations:** National Eye Institute, National Institutes of Health, Bethesda, MD 20892 USA

## Abstract

Visualizing the cellular manifestation of disease has recently been aided by an increasing number of adaptive optics (AO)-based imaging modalities developed for the living human eye. However, simultaneous visualization of multiple, interacting cell types within a complete neural–epithelial–vascular complex has proven challenging. By incorporating AO with indocyanine green angiography, we demonstrate the possibility of imaging photoreceptors, retinal pigment epithelial cells, and choriocapillaris in the living human eye. Unexpectedly, we found that there was uptake of indocyanine green dye into the retinal pigment epithelial cells in the earliest phases of imaging, which formed the basis for devising a strategy to visualize the choriocapillaris. Our results expand the range of applications for an existing, FDA-approved, systemically injected fluorescent dye. The combined multimodal approach can be used to evaluate the complete outer retinal complex at the cellular level, a transformative step toward revealing the in vivo cellular status of neurodegenerative conditions and blinding diseases.

## Abstract (web summary)

HaeWon Jung, Tao Liu et al. combine multimodal adaptive optics with indocyanine green angiography to improve imaging of the human eye. This combination allowed visualization of photoreceptors, retinal pigment epithelial cells, and choriocapillaris of the living eye with remarkable resolution.

## Introduction

Neurodegenerative diseases cause some of the most debilitating disabilities in modern society, and this is exacerbated by the fact that there are very few options available for treatments to cure or delay the onset of disease. By the time disease is detected, dramatic changes to the cellular environment have already occurred. These complex cell-to-cell interactions and corresponding sequence of events leading to degeneration are difficult to capture and recapitulate by ex vivo models of human disease, making development and testing of potential treatments a particularly challenging and expensive endeavor. One way to investigate neurodegenerative diseases in vivo is to consider the photoreceptor (PR)–retinal pigment epithelial (RPE)–choriocapillaris (PR–RPE–CC) complex of the human eye. This is a multilayered homeostatic unit critical for human vision consisting of neurons, epithelial cells, and a microvasculature. Disruption to this unit has been associated with a wide range of diseases, including age-related macular degeneration^1,2^, inherited retinal degenerations^2,3^, Alzheimer disease^4^, and even atherosclerosis^5^.

Indocyanine green (ICG) angiography (ICGA) is routinely used in the eye clinic for ophthalmic angiography, and is the gold standard for imaging the choroidal vasculature, largely because it is almost completely protein-bound and therefore thought to be retained in the choriocapillaris, in addition to the fact that ICG fluoresces in the near-infrared wavelengths, which penetrates deeper into the posterior portions of the eye^6^. However, it has not been straightforward to use ICGA to visualize the choriocapillaris^7,8^ (the microvasculature of the outer retina) and currently ICGA is considered to be suited for imaging only larger choroidal vessels^9^. Imaging of the choriocapillaris based on optical coherence tomography angiography has demonstrated promising results^10–16^, but it remains challenging to clearly resolve choriocapillaris and flow voids in some areas of the eye, such as the foveal center.

In this paper, we sought to improve the gold standard for imaging choroidal vessels by incorporating adaptive optics (AO) with ICG for the purposes of angiography (AO-ICGA), and to illustrate a potential clinical application. Due to the improved signal and resolution afforded by AO, we also show that there is rapid uptake of ICG dye into the overlying RPE cells in the earliest phases of ICG imaging, which is an intriguing phenomenon that has not been previously explored. When combined with additional multimodal AO methodologies, we demonstrate visualization of the entire PR–RPE–CC complex in the living human eye.

## Results

**Fig. 1:**
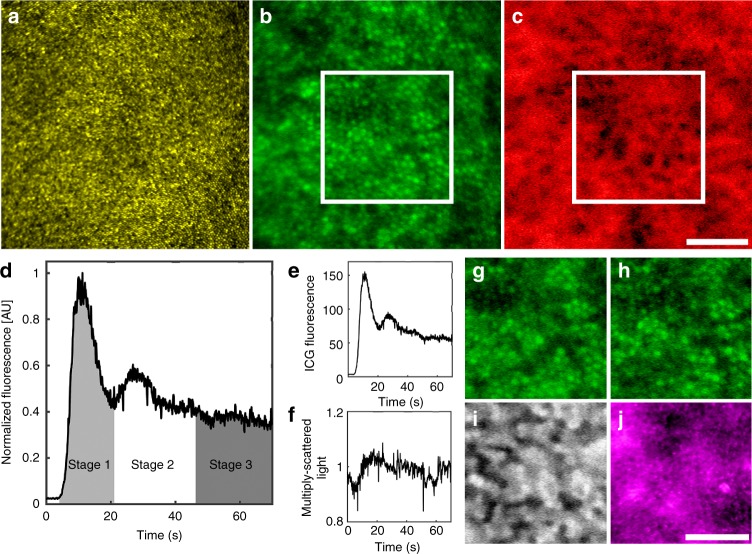
Multimodal AO imaging of the PR–RPE–CC complex in the living human eye (subject 15 from Supplementary Table 2). **a** Image of cone photoreceptors which appear as tiny, bright punctate spots of varying intensity (backscattered light captured by the confocal reflectance channel^56,61^). **b** Fluorescence image of RPE cells captured in the initial minute of AO-ICGA (based on ref. ^18^). **c** Image of the choriocapillaris microvasculature computed from AO-ICG images. **d** Temporal dynamics of AO-ICG signal in the first minute following systemic injection of ICG dye showing three stages (normalized for fluctuations in signal intensity detected in a nonfluorescent channel). The relative intensity level of the background near-infrared autofluorescence^18,32^ can be seen in the initial tail of the plot (first few seconds) before the dye bolus arrives. **e** AO-ICG signal before normalization. **f** Multiply scattered light imaging channel used to normalize the AO-ICG signal (the multiply scattered light channel itself is first normalized around one, as shown here). **g–j** Zoom of boxes in **b**, **c** as seen by various channels. **g**, **h** AO-ICG signal corresponding to stage 1 and stage 3, respectively. Subtraction of (h) from (g) reveals the choriocapillaris (box in (**c**)). (i) The same retinal area as **g**, **h** imaged in the late phase (134 min afterwards), showing a characteristic heterogeneous pattern of fluorescence^18^. **j** Image of outlines of RPE cells based on simultaneously captured darkfield images (backscattered, nonfluorescent light^19^). All scale bars, 100 µm

Similar to the case of AO-based fluorescein angiography^17^, our implementation of AO-enhanced ICG (AO-ICG) imaging^18^ successfully revealed the inner retinal microcirculation, establishing that our AO system was capable of detecting ICG fluorescent light (Supplementary Figure 1). Although AO improved the quality and resolution of ICGA (Fig. 1 and Supplementary Figure 1), unfortunately, it was not straightforward to directly visualize the choriocapillaris in a similar manner. By combining both backscattered and fluorescent light from different timepoints, we devised a strategy for visualizing the entire PR–RPE–CC complex based on the uptake of ICG dye combined with high-resolution retinal imaging enabled by our custom-built multimodal AO retinal imager. We also derived a quantitative method to capture the overall health of the systemic vasculature represented by the ophthalmic vasculature, based on the temporal dynamics of the ICG fluorescence signal immediately following injection (Fig. 1d), which we describe below. These techniques will form an important part of the suite of multimodal AO retinal imaging tools (color scheme defined in Supplementary Table 1) that are available for assessing the structure and function of the human retina at cellular-level resolution.

## Imaging RPE cells in the early phases of ICGA

Unexpectedly, ICG dye was taken up by the RPE cells immediately upon entering the eye (Fig. 1b), which provided a method to image RPE cells in the living human eye. The uptake of ICG dye into a subset of RPE cells appeared to be a relatively uniform event, in that ICG-labeled cells exhibited similar levels of fluorescence, in sharp contrast to the heterogeneous images of RPE cells observed in the late phase of AO-ICG imaging (Fig. 1i) as we showed previously (between 10 and 20 min and several hours after injection^18^). Interestingly, ICG was taken up by both RPE cells that were directly above choriocapillaris vessel segments as well as by those that were adjacent to these. To validate that the fluorescent cells that we were observing were in fact RPE cells, we compared early phase AO-ICG images with simultaneously acquired, co-registered darkfield images of the same retinal regions which showed outlines of RPE cells (darkfield was previously validated to originate from the RPE^19^). Qualitatively, we found that RPE cells were more easily visualized using the ICG method than darkfield (Supplementary Figure 2). For the purposes of this validation, we restricted our analysis to only those subjects with high quality darkfield images in which RPE cells could be unambiguously identified (which restricted the analysis to only 4 out of 17 possible eyes; Supplementary Table 2). In this subset of subjects, there was good colocalization of identified RPE cells across the two modalities (Supplementary Figure 3). Quantitative comparison of cells identified across the two co-registered modalities revealed that there was good agreement between pairs of images with an overall recall of 0.91 ± 0.05 and precision of 0.95 ± 0.04 (*n* = 328 RPE cells; Supplementary Table 3). The higher number of false negatives (i.e., RPE cells seen on darkfield but not on AO-ICG) compared to false positives is due to the fact that there are some RPE cells (a small subset) that do not appear to take up ICG dye in the early phase, which we did not identify for the purposes of this comparison. However, the precision was high, indicating a high likelihood that ICG-labeled cells were RPE. Given the overall difficulty of robustly imaging RPE cells in the living human eye, we expect that the early phase AO-ICG approach will contribute to the suite of multimodal strategies needed to reliably assess the RPE across larger patient populations.

The observation that there was rapid uptake of ICG dye into the RPE in the early phase following systemic injection was further corroborated by AO-ICG images of the inner retinal vasculature that we acquired during the first five minutes following dye injection (Supplementary Figure 4). When using a larger confocal aperture (leading to a larger effective depth of focus), out of focus fluorescent light from the underlying RPE mosaic was visible in the background. We subsequently confirmed in a follow up visit (after the dye cleared from the eye) with a repeat injection of ICG dye that the use of a smaller confocal pinhole (better optical sectioning) rejected the majority of the out of focus light from the RPE. This data confirms that the signal that we are observing arises from the outer retina and is not the result of out of focus light from the inner retina.

## Imaging the choriocapillaris using AO-ICGA

The immediate uptake of ICG dye into RPE cells explains why the choriocapillaris was not previously resolvable by conventional ICGA unless sequentially acquired images were subtracted from each other^7^. Here, we show that removal of the RPE signal is a key enabling step for improving AO-ICGA imaging of the choriocapillaris.

**Fig. 2:**
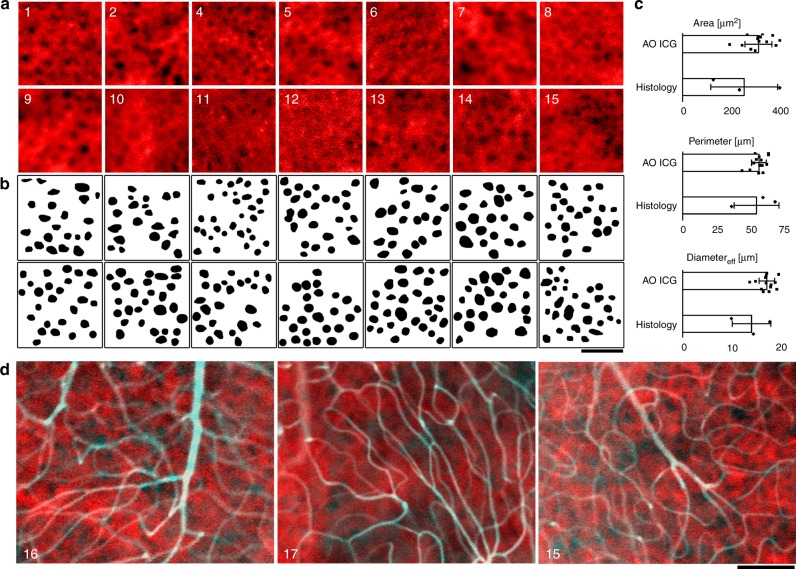
Visualization of choriocapillaris in healthy eyes. **a** Each panel represents an image of foveal choriocapillaris from a different subject (subject number indicated in top left corner). In the fovea, there are no inner retinal vessels present. **b** Semiautomated segmentation of identifiable flow voids corresponding to (**a**). **c** Morphometric comparison of AO-ICG images of choriocapillaris flow voids (*n* = 14 subjects) to published histology from macular regions of human donor eyes (*n* = 3 subjects) (error bar, SD). Source data provided in Supplementary Data 1. **d** Sequentially acquired images of choriocapillaris (red) and inner retinal vessels (cyan) at nonfoveal regions, at retinal eccentricities of 0.9, 1.4, and 1.6 mm (from left to right). All scale bars, 100 µm

Although the fluorescence intensity of RPE cells in the early phase of AO-ICG imaging appears to be fairly uniform, there were some time-dependent variations in the lower-spatial-frequency hyper- and hypofluorescent patterns present (Fig. 1g, h), which we anticipated might arise from the underlying choriocapillaris. In a subset of subjects in which we acquired images of ICG dye passage at the fovea (Supplementary Table 2), we devised a strategy to remove the RPE signal from the stage 1 (Fig. 1d) AO-ICG images in order to reveal the underlying choriocapillaris. We found that the injection of a bolus of ICG dye was detectable by following the rise and fall of the ICG fluorescence intensity over time with a clear peak corresponding to the initial entry and exit of the bolus of dye in the vasculature. Based on the location of this primary peak, we surmised that both stage 1 and stage 3 images contained signal from both RPE and choriocapillaris, but that stage 1 was more likely to contain relatively higher levels of choriocapillaris signal, whereas stage 3 was more likely to contain relatively higher levels of RPE signal. Subtracting stage 3 (Fig. 1h) from stage 1 (Fig. 1g) effectively removed the RPE signal and revealed the underlying choriocapillaris (box in Fig. 1c). We accounted for fluctuations in fluorescence intensity arising from subject- or instrument-related issues by normalizing AO-ICG signals (see Methods) (Fig. 1e, f). Using this strategy, we were able to visualize foveal choriocapillaris in a majority of subjects (14 out of 17). Unsuccessful images were primarily due to poor image quality during stage 1. When stage 1 images were of high quality, we were able to obtain successful images with the start of stage 3 empirically determined to be on average 59 s after the peak of stage 1 (the time duration of stage 3 was on average 47 s, ranging from 16 to 102 s). Importantly, at the fovea, we expected that the AO-ICG signal arose from only RPE and choriocapillaris (no overlying inner retinal vessels due to the foveal avascular zone which is identifiable by AO imaging^20,21^). Quantification of manually segmented foveal choriocapillaris flow voids in these eyes was within the range of histological measurements based on performing the same quantitative analysis of flow voids on published data^22–24^ (no significant differences in area (*p* = 0.54), perimeter (*p* = 0.87), or effective diameter (*p* = 0.30) between AO-ICG and histology) (two-tailed, unpooled *t* test for all tests here; see Fig. 2a–c, Supplementary Table 4). Taking the two-dimensional power spectra of this data revealed an average row-to-row spacing (average spacing between choriocapillaris flow voids) of 30.9 ± 5.2 µm (mean ± SD; see Supplementary Table 5), which was similar to a previously reported measurement of 39 µm from a single foveal choriocapillaris image generated using adaptive optics–optical coherence tomography (AO-OCT)^12^ (taken a few degrees temporal to the fovea; published AO-OCT data at the fovea was not available).

**Fig. 3:**
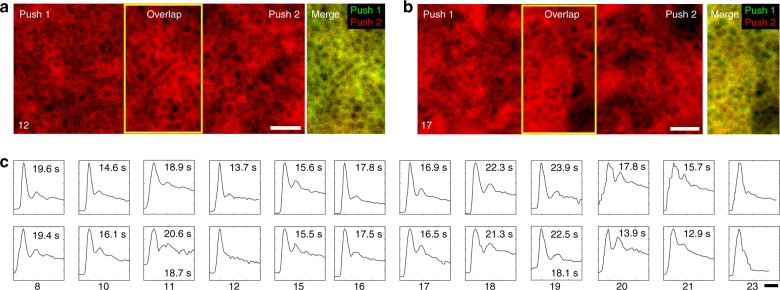
Repeatability of choriocapillaris visualization and recirculation time measurements. **a**, **b** Extending the effective field of view using sequentially acquired, overlapping (**a**) foveal choriocapillaris images and (**b**) nonfoveal choriocapillaris images (retinal eccentricity: 1.2 mm). There was good agreement between choriocapillaris visualized in overlapping regions between two pushes (yellow boxes; color-merged images on right with push 1 in red, push 2 in green). Subject numbers are given in the bottom left hand corner. Scale bars, 100 µm. **c** The AO-ICG fluorescence intensity time plot (*x*-axis: time in seconds, *y*-axis: AO-ICG fluorescence intensity, arbitrary units) was highly repeatable and appears to be characteristic to the subject being imaged (each column is one subject, indicated on the bottom). First pushes are shown in the top row; second pushes, bottom row. Recirculation times corresponding to each plot are listed (seconds; some were not measurable as described in the text). Subjects 11 and 19 were given three pushes (1 + 1 + 1; recirculation time shown below; plots corresponding to third pushes are shown in Supplementary Figure 6). Subject 23 was imaged at a deep focal plane posterior to the choriocapillaris and it is unclear whether there is a second peak present. Scale bar, 20 s

We were also able to successfully visualize choriocapillaris at nonfoveal regions of the eye (Fig. 2d, Fig. 3b) by following the same strategy. Sequentially acquired images at an anterior focus confirmed that the choriocapillaris signal was distinct from signals arising from the inner retinal vessels. Changing the focus towards the posterior direction also revealed larger choroidal vessels which were otherwise blocked by the ICG fluorescence within the RPE cells (Supplementary Figure 5). In most of our images, we set our focal plane slightly posterior to the best focal plane for photoreceptor imaging (while still allowing for reasonable images of photoreceptors). Future optimization of focusing may lead to additional improvements in image quality. Nevertheless, our initial results open up a new possibility for visualizing the PR–RPE–CC complex and surrounding vasculature.

## Repeatability and measures of vascular perfusion health

Given that one of the main limitations of the technique was the restriction of imaging the choriocapillaris at only one location per injection, we devised a strategy in which we divided the total volume of the ICG dye bolus into multiple injections, which could be given sequentially (at the cost of weaker signal). These sequential injections enabled different locations of the eye to be imaged for the purposes of enlarging the area imaged (Fig. 3a, b) or also for acquiring images in contralateral eyes. Despite the presence of ICG dye in the RPE cells prior to the administration of additional injections (due to uptake from the initial injection), our strategy for subtracting the RPE signal was still able to visualize choriocapillaris, partly due to our choice of unevenly splitting the bolus (1 + 2 mL), which allowed the second injection to have higher signal than the first. In an overlapping area between two injections, images of choriocapillaris were repeatable, with good colocalization of signal in regions that overlapped between two pushes (Fig. 3a, b).

In addition to a primary peak arising from the passage of the initial bolus of ICG dye passing through the eye, we could detect a secondary peak of lower intensity (Fig. 3C) that we interpret to arise from recirculation of the initial ICG dye bolus back through the eye. The broadening and weakening of the secondary peak compared to the primary peak is likely due to local mixing of the ICG dye bolus with the bloodstream. The time between peaks was similar across subjects: 18.31 ± 2.98 s (mean ± SD, calculated from the first push of 16 subjects, Supplementary Table 1). The unmeasurable data was mostly due to inopportune blinks or issues with data acquisition that coincided exactly with the location of either peak (Supplementary Figure 6), or in one case, due to changing the focus to a deeper choroidal vessel. Although the passage and recirculation of a bolus of dye through the eye has not been previously visualized, our measurements are similar to a simulation of a bolus of ICG dye recirculating through the aorta (~25 s)^25^. Remarkably, the shape of the AO-ICG fluorescence intensity time plot was highly repeatable within the same subject when comparing plots from sequential injections (Fig. 3c). Although additional injections were given while imaging in different parts of the retina, the corresponding shape and measured recirculation times were still preserved (Fig. 3c, *p* = 0.22, two-tailed, paired *t* test), suggesting that this curve is a measure of overall perfusion health of the systemic vasculature that is influenced by the ocular choroidal vasculature. In the future, unraveling the biological consequences of differences in the shapes of the AO-ICG fluorescence intensity time plots may lead to new insights about the function of the systemic vasculature as seen through the eye. Our preliminary measurements of recirculation time may be useful as a biomarker for vascular health.

## Multimodal AO imaging of the complete PR–RPE–CC complex

**Fig. 4:**
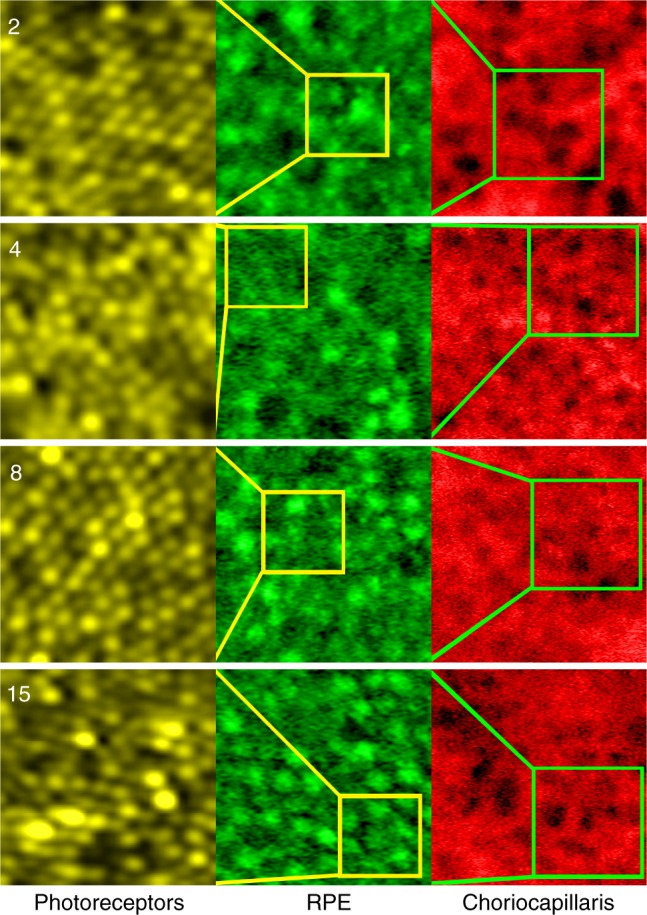
Size scale comparison of foveal cones, RPE cells, and choriocapillaris. Each row represents data from one subject (codes indicated in top left corners). Each column represents a different zoom: the photoreceptor image (yellow, 37 µm x 37 µm ROI) is taken from a region corresponding to the yellow box in the RPE image (green, 100 µm × 100 µm ROI), which is taken from a region corresponding to the green box in the choriocapillaris image (red, 200 µm × 200 µm ROI). The AO-ICG image of RPE cells is enhanced for visualization purposes (see Methods)

The combination of fluorescent and nonfluorescent AO imaging modalities enabled the visualization of the entire PR–RPE–CC complex in the living human eye. Direct measurement of the size scale of simultaneously acquired, co-registered foveal images revealed that the size scale of the RPE and choriocapillaris flow voids were on average 3.1 and 3.7 times larger than the size scale of overlying cone photoreceptors, respectively (Fig. 4, Supplementary Table 6). These relative size differences support the notion that we are faithfully visualizing the PR–RPE–CC complex and provides a direct quantitative measure of each layer. All in all, direct comparison of simultaneously acquired images of the neurons, surrounding cells, and vasculature (Fig. 4, Supplementary Figure 1) provides a tool for assessing the status of neural tissue at the cellular level that has important implications for measuring disease-related change in patients with neurodegenerative or blinding retinal diseases.

## Clinical application in a patient with retinitis pigmentosa

**Fig. 5:**
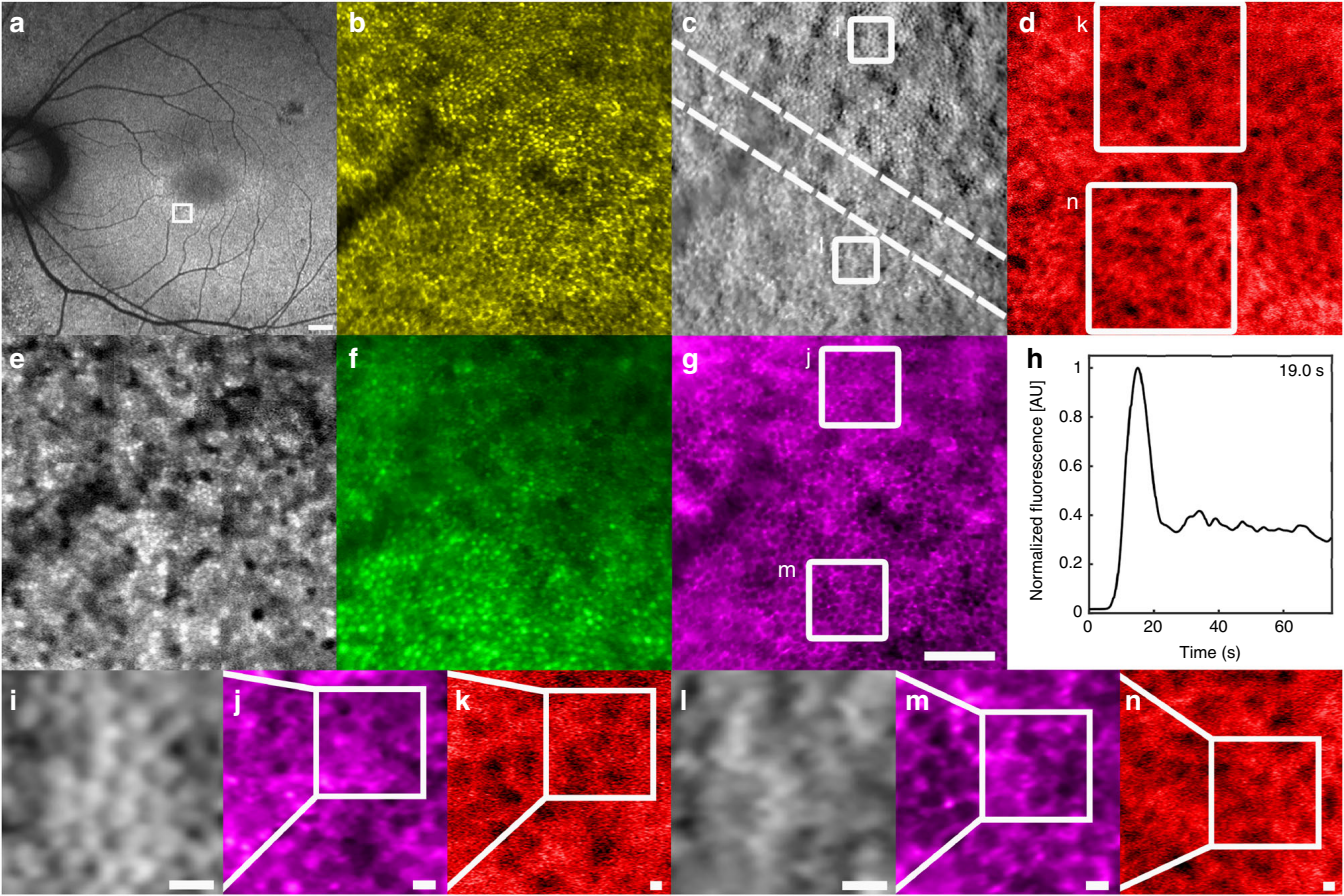
Clinical application of multimodal AO retinal imaging to assess the transition zone of a patient with retinitis pigmentosa. **a** Fundus autofluorescence image acquired using a commercially available instrument (Optos Panoramic Ophthalmoscope—P200MAAF (200Tx), model A10092, Scotland, UK). White box indicates the area where AO images were acquired. Scale bar, 500 µm. **b**, **c** Intact cone photoreceptors were observed (upper right corner) with a rapid transition to RPE cells (outlines of RPE cells visible in the lower left corner) in **b** confocal reflectance and **c** split detection images.The approximate start and end of the transition zone is marked with dashed lines. Both the **d** choriocapillaris and the **e**–**g** RPE cells were intact and well-preserved across the entire transition zone. There is some difference in the fluorescence of RPE cells in **f** which may be due to the presence/absence of overlying photoreceptor cells (cone imprinting^18^, also seen in **e**). **h** ICG fluorescence time plot from which recirculation time was measured. Scale bar for **b**–**g**, 100 µm. **i**–**n** Zooms of ROIs 1 and 2 marked in white boxes in **c**, **d**, and **g** (note that **i** is a zoom of the location indicated by the white box in **j** which is a zoom of the location indicated by the white box in **k**; similarly **l** is a zoom of the white box in **m** which is a zoom of the white box in **n**). **i**–**k** ROI 1, an area with intact cone photoreceptors. (**l**–**n**) ROI 2, an area with no cone photoreceptors but intact RPE. The sizes of the ROIs are **i**, **l** 50 µm, **j**, **m** 100 µm, and **k**, **n** 200 µm. Scale bar for **i**–**n**, 10 µm

To demonstrate the value of our multimodal approach, we imaged a patient with nonsyndromic retinitis pigmentosa at a transition zone between healthy and diseased retina. Conventional clinical tests revealed that there was retinal atrophy and photoreceptor loss with an island of preservation at the fovea. There was a hyper-autofluorescent ring surrounding the fovea suggestive of RPE dysfunction (Fig. 5a). Although AO has been previously applied to examine patients with RP, with loss of photoreceptors demonstrated in a number of studies^26–28^, the status of the underlying RPE and choriocapillaris remains less clear. In some patients, intact RPE can be seen using confocal reflectance light in areas where photoreceptors have been lost^29^. Visualizing these cells from a multimodal perspective could help confirm the layer-specific consequences of disease, a critical first step towards developing treatments tailored toward neuronal, epithelial, or vascular health to preserve remaining cells during disease.

Multimodal AO imaging confirmed that there was an island of preserved photoreceptors with an abrupt transition from intact, contiguous photoreceptors (Fig. 5b, c: upper right) to what appeared to be complete loss of photoreceptors (Fig. 5b, c: lower left). As has been previously reported, we were able to visualize RPE cells using confocal reflectance in areas where overlying photoreceptors were absent^29^. In an area near the boundary of photoreceptor atrophy, where photoreceptors are still present, the cone spacing was 8.8 µm (0.75 mm eccentricity from the fovea) (Supplementary Table 6), which is within the expected range of spacing (or very slightly higher)^30,31^, suggesting that cone photoreceptor inner segments are fairly well preserved up to the edge of atrophy in this patient. To our knowledge, we show for the first time a total of five types of AO images (Fig. 5b, c, e–g) that together confirm the relative preservation of the RPE mosaic across the area of the transition zone that was imaged. The RPE cell-to-cell spacing measured at two ROIs across the transition zone (13.2 and 14.9 µm; Supplementary Table 6) were within normal ranges^32^, further supporting the notion that RPE cells are well-preserved. Interestingly, there was rapid uptake of ICG dye into RPE cells in the areas that appeared to lack photoreceptors in our multimodal AO images. Structurally, the underlying choriocapillaris was also intact across the entire transition zone and quantified flow void sizes were similar to the normal range (Supplementary Tables 4 and 6). The recirculation time in this patient was measured to be 19.0 s which was within the expected normal range (*z*-score: 0.23). Together, this data provides a powerful tool for revealing the cellular status of disease in the living human eye, showing that in this eye, there is abrupt loss of photoreceptors in the context of relatively well-preserved RPE and choriocapillaris across the entire transition zone.

## Discussion

Our understanding of cellular changes in situ is often restricted to biopsies and postmortem tissue, where it is too late to unravel the layer-specific sequence of cellular events that leads to disease. Identifying the manifestation and consequences of disease at the cellular level and at the very earliest stages is a critical first step towards developing better treatments. The multimodal combination of seven different types of AO images (Supplementary Table 1) acquired in a patient with retinitis pigmentosa demonstrates abrupt loss of neurons at the disease front with preservation of the underlying epithelial and vascular tissue, providing an important tool for evaluating the status of different classes of cells in an area of active disease process.

Our results improve the gold-standard for choroidal angiography in the eye, not only with the visualization of the smallest choroidal microvascular structures, but also with the capability to assess the functional health of the systemic vascular perfusion. Interestingly, there was a rapid uptake of ICG dye into the overlying RPE cells, a direct visualization of uptake of an extrinsic agent in the outer blood retinal barrier (analogous to the blood brain barrier)—further characterization of this phenomenon may lead to new insights about the pharmacokinetics of drug delivery in the outer retina. Visualization of the dynamic interaction between an extrinsic dye and the RPE provides complementary information to the growing list of established AO-based techniques for imaging the RPE, based on detection of multiply scattered light (e.g., darkfield^19^), autofluorescent light (e.g., visible^33^ and near-infrared^32^ autofluorescence), and interferometric light (e.g., optical coherence tomography^34^). The main advantage of our technique is that it provides a more objective method to probe the outer retinal complex as it does not rely on intrinsic sources of contrast, which are likely to change due to disease processes. In contrast to our related method in which we image ICG dye uptake into the RPE in the later phases^18^, the approach that we demonstrate in this manuscript has the advantage that the RPE cells which become labeled with ICG dye are more uniformly fluorescent.

A major limitation of the AO-ICG technique for imaging choriocapillaris is the relatively small field of view (this is a fundamental limitation of AO technology that is rooted in the finite size of the isoplanatic patch—typically one to two degrees of visual angle). Future improvements in AO instrumentation may lead to the possibility to explore AO-ICG imaging of choriocapillaris with larger fields of view^35,36^ and with even smaller amounts of ICG, as has been previously demonstrated for visualization of feeder vessels^37^. Measurements from our data indicate an average size scale for the choriocapillaris of 15–30 µm, suggesting that it may be possible to generalize our strategy to visualize the choriocapillaris using instruments that do not utilize AO, such as a scanning laser ophthalmoscope^38^ that has a much larger field of view than can be achieved using adaptive optics scanning laser ophthalmoscopes (AOSLO). Even with our limited field of view, we expect that the AO-ICG technique will be insightful for evaluating transition zones of diseases when combined with other modalities (Fig. 5). In particular, the combination of our AO-ICG technique with other AO modalities such as AO-OCT^39–43^ will open up the possibility to visualize the structure and function of the vasculature from multiple perspectives. Recent improvements in optical coherence tomography angiography (OCTA)^10,11,44–46^, including AO-OCTA^47–51^, have now lead to alternative approaches to image the choriocapillaris in the living human eye. The advantages of OCTA are that it is rapid, noninvasive, and inherently a three-dimensional method to visualize the chorioretinal vasculature; the advantages of AO-ICG and other AOSLO-based angiography techniques^17,20,21,52–55^ lie in their ability to directly visualize perfusion (including leakage in the case of AO-ICG, which cannot be detected using OCTA). To our knowledge, the AO-ICG technique proposed in this paper represents the first demonstration of an AOSLO-based technique for visualizing choroidal vessels. In the future, direct comparisons between these techniques will be insightful and necessary for further cross-validation.

All in all, this approach opens up exciting possibilities for the quantification of novel, more sensitive neural, epithelial, and vascular biomarkers of cellular and systemic health to track disease onset and progression using the eye as a window to the central nervous system and systemic vasculature. We demonstrate an application of AO-ICG that provides an unexpected opportunity to visualize a different aspect of the RPE in addition to vasculature. Further investigation of the dynamics and mechanisms of ICG uptake from the vasculature into the RPE could serve as a model for characterizing drug delivery to the eye in the context of the outer blood retinal barrier (analogous to the blood brain barrier), or could also lead to functional measurements of the health of the RPE-choriocapillaris interface based on the amount (or rate) of dye transferred from the choriocapillaris to the RPE (e.g., Bruch’s membrane permeability, thought to be affected in disease).

## Methods

### Human subjects and clinical evaluation

Healthy subjects with visual acuity of 20/20 or better, no history or signs of ocular disease in at least one eye, and no known allergies to shellfish, iodine, or indocyanine green were recruited for this study (NCT02317328). All patients underwent comprehensive ophthalmic assessment including dilated examination. A total of 23 healthy subjects (age 31.7 ± 9.4 years, 9 male/14 female) were included in the study (Supplementary Table 2). One 49-year-old female patient with nonsyndromic retinitis pigmentosa was included in this analysis. This study was approved by the Institutional Review Board of the National Institutes of Health. Research procedures adhere to the tenets of the Declaration of Helsinki. Written, informed consent was obtained from all participants after the nature of the research and possible consequences of the study were explained.

### Experimental design

Eyes were dilated with 2.5% phenylephrine hydrochloride and 1% tropicamide, and then AO retinal imaging was performed using custom-assembled multimodal instrument^56,57^ modified to be capable of detecting ICG fluorescence^18^ (see Multimodal AO retinal imager, below). Images were acquired before, during, and after intravenous administration of a total ICG dose of 25 mg in 3 mL, according to the standard of care at the National Eye Institute Eye Clinic. Subjects were asked to look at a fixation target and were allowed to blink naturally throughout the data acquisition. Depending on the aims being investigated, different retinal locations and focal depths were imaged (Supplementary Table 2). With the exception of one dataset (marked in Supplementary Table 2), the focal plane selected for image acquisition was set to be near the plane of best focus for the confocal reflectance image of the photoreceptors, as has been previously reported for photoreceptor/RPE imaging^18,19,32^ (it is not surprising that the entire complex can be captured with one focal plane given that the RPE-Bruch’s Membrane–Choriocapillaris complex is approximately similar to the theoretical depth of focus of our instrument, ~27 µm). Images were corrected for eye motion^58^ based on the eye motion correction computed from one of the simultaneously acquired nonfluorescent channels^18^. Registered, averaged images from different time points were used to visualize the PR–RPE–CC complex. Fluorescence intensity time plots were normalized and used to quantify recirculation time.

### Statistical analysis

Flow void measurements from AO-ICG and histology were compared using a two-tailed, unpooled *t* test; recirculation times from first and second pushes were compared using a two-tailed, paired *t* test.

### Multimodal AO retinal imager

The custom-assembled AO retinal imager^56^ incorporated split detection^57^, darkfield^19^, and AO-ICG^18^ channels and was outfitted with a computer-controlled fixation system^59^. Subjects were imaged using multiple versions of instrumentation for detection of AO-ICG signal, including the initial implementation^18^, a second previously described improved implementation^32^, and a third further improved system, which we describe here. In the first two implementations, ICG fluorescent light was collected between 810 and 830 nm using a standard (noncustom) dichroic (LPD02-830RU, Semrock, Rochester, NY, USA) following excitation with a broadband 790 nm light source (~17 nm full width half max); wavefront sensing was performed using an 850 nm light source. In the improved second implementation, the 790 nm excitation light source power measured at the cornea was increased from 100 to 115–135 µW and the fixation pellicle used in the initial implementation (~90% transmission of 750–850 nm light) was replaced with a dichroic beamsplitter (>95% transmission of 750–850 nm light) to allow for more efficient delivery of excitation light and more efficient detection of fluorescent ICG light. In the third implementation, we replaced the standard dichroic with a custom 855 nm dichroic (Semrock, Rochester, NY, USA) to effectively collect light between 810 and 850 nm which required wavefront sensing to be performed at 880 nm (Superlum SLD-mCS-341-HP1-SM-880, Ireland) with clean-up filters (Semrock FF01–900/32, Rochester, NY, USA) placed in front of both the light source and the wavefront sensor. The size of the confocal aperture placed in front of the ICG detection arm ranged from 2.5 to 8.0 Airy disk diameters (approximate lateral resolution: 2–3 µm, depending on the axial length of the eye imaged). The power of the light measured at the cornea was maintained below 135 µW for the 790 nm excitation light source and 40 µW for the 880 nm wavefront sensing light source (35 µW for the 850 nm wavefront sensing light source for the initial two implementations). These light levels were below the maximum permissible exposure limits set by the American National Standards Institute standard Z136.1 2014.

### Validation of RPE imaging using early phase AO-ICG images

AO-ICG images generated by averaging the first 40–80 s of eye-motion-corrected frames were compared to simultaneously captured and co-registered darkfield images. In the subset of subjects who had high quality darkfield images of RPE cell outlines, RPE cell centroids were manually and independently identified in each pair of AO-ICG/darkfield images and then compared to each other to quantify the numbers of true and false positives and negatives^30^, where the threshold for determining correspondence between markings was set to half of the measured RPE cell-to-cell spacing from the darkfield image averaged across the subjects. All of this analysis was done by a single expert grader (H.J.) and the two sets of images were independently analyzed before the comparisons were made. For visualization purposes (Fig. 4, Supplementary Figure 3), AO-ICG and darkfield images were high pass filtered (based on the measured size of RPE cells) and contrast stretched.

### Choriocapillaris imaging and recirculation time measurement

AO-ICG video frames were registered to a single reference frame with minimal distortion^58^ and then normalized either to itself (autonormalization), for the purposes of generating choriocapillaris images, or to a simultaneously acquired nonfluorescent (normalization) channel, for the purposes of measuring recirculation time. Imaging data corresponding to blinks were discarded. For autonormalization, we automatically detected a small subset of the most hypofluorescent flow voids (an indirect measure of the background signal), and then used the average fluorescence measured in this subset of dark flow voids to normalize the AO-ICG video frames. For normalization, we divided each AO-ICG frame by a factor linearly proportional to the intensity of a nonfluorescent multiply scattered light channel to rescale the intensity values from 0 to 1. In this initial implementation, we only computed choriocapillaris images from an ROI contained within the frames that contained signal across the entire video (due to eye motion, some pixels at the edges are only seen in a smaller subset of frames; these edge pixels were discarded). We defined stage 1 as the time interval corresponding to the first peak, and stage 3 as a time interval beyond the second peak after which the signal becomes relatively stable (in the case of third or fourth peaks existing, the time window for stage 3 was shifted beyond the last visible peak). Finally, AO-ICG images averaged across the time windows for stage 3 were subtracted from those corresponding to only the rising phase of stage 1 (which contained higher signal than the falling phase of stage 1) to generate the final choriocapillaris image. To measure recirculation time, following normalization of the AO-ICG signal, gaussian filtering was applied to smooth the signal in the time dimension (filter window: 15 frames) to improve the robustness of detecting local maxima, and the time between peaks was measured.

### Quantification of cell spacing and flow voids

Photoreceptors and RPE cells were manually identified and the coordinates of identifications used to compute average cell-to-cell spacing based on the density recovery profile as previously described^26,30^. For quantification of choriocapillaris flow voids, following application of the Frangi filter^60^, images of choriocapillaris were binarized using a manually determined threshold. The preliminary binary mask was then overlaid on the original choriocapillaris image and each segmented flow void was manually adjusted to correct for any errors in segmentation. Segmentations that did not appear to arise from actual flow voids were discarded. All segmentations were performed by the same expert grader (H.J.) for consistency. Following segmentation, pixel scaling information was determined and used to quantify areas, perimeters, and effective diameters. For AO-ICG data, pixels were converted to micrometers as previously described^30^. At the fovea, we used 37 µm × 37 µm ROIs for quantifying cone photoreceptors, 100 µm × 100 µm ROIs for quantifying RPE cells and 200 µm × 200 µm ROIs for quantifying choriocapillaris flow voids. For the patient with retinitis pigmentosa, we used a 50 µm × 50 µm ROI for quantification of photoreceptors at the border of photoreceptor atrophy. Segmentation of flow voids from published histological data was performed manually and then quantified in the same manner, based on the reported scaling information. The two-dimensional power spectrum analysis was performed as previously described^12^. Briefly, for each subject, the AO-ICG choriocapillaris image was cropped to 500 pixels × 500 pixels in its native pixel sampling (unique for each subject due to their biometric data) and then calculated by taking the two-dimensional fast Fourier transform of the image and multiplying it by its complex conjugate. The power spectral density was radially averaged to give a one-dimensional profile and used to identify peak spatial frequencies.

### Code availability statement

Custom codes which have not been previously described in the published literature used in this research will be made available by the corresponding author upon reasonable request.

## Electronic supplementary material

Supplementary Information

Supplementary Data

Description of additional supplementary items

## Data Availability

The data that support the findings of this study are available from the corresponding author upon reasonable request.
