# Supplementary material for: Combining multimodal adaptive optics imaging and angiography improves visualization of human eyes with cellular-level resolution
Source: Commun Biol. 2018 Nov 14;1:189. doi: 10.1038/s42003-018-0190-8 (PMC6235967; doi:10.1038/s42003-018-0190-8)
Supplement: Supplementary file 1 — Supplementary Information [file 42003_2018_190_MOESM1_ESM.pdf]

Supplementary Information

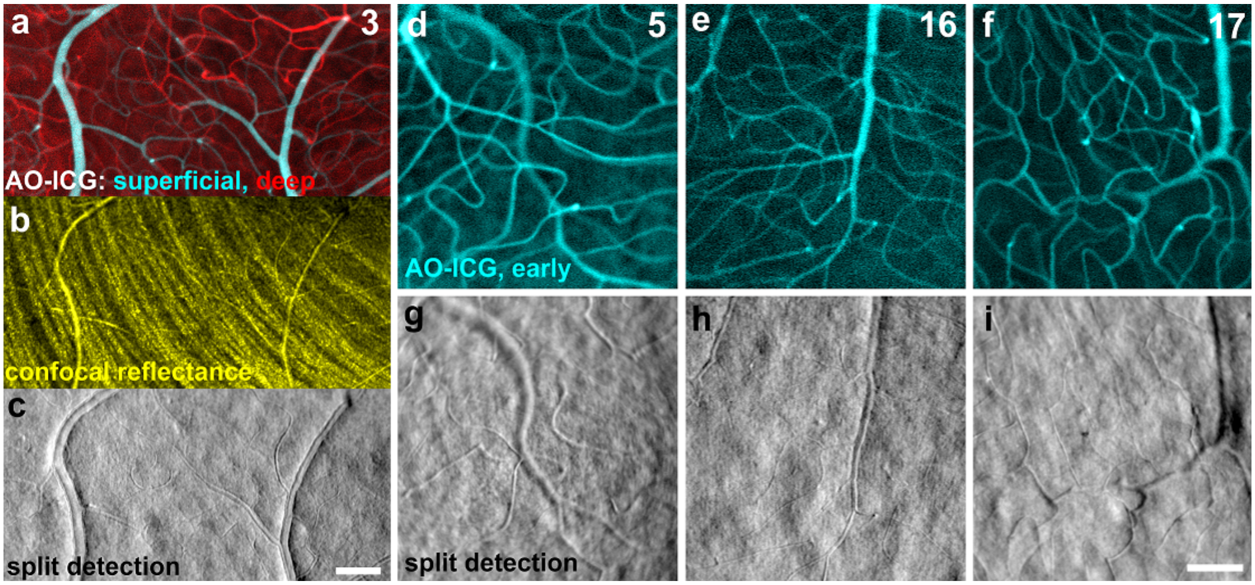

Supplementary Figure 1. Multimodal AO imaging of inner retinal vessels. (a-c) Inner retinal neurovascular complex. (a) Superficial vessels (cyan) and deep capillary plexus (red) acquired sequentially using AO-ICGA. (b) Ganglion cell axon bundles (striations) acquired using confocal reflectance light. Some blood vessels can be seen. (c) Vessel walls imaged using split detection. Superficial vessels in (a) were acquired simultaneously alongside (b) and (c). (d-f) AO-ICG images of the inner retinal vasculature. (g-i) Simultaneously-acquired split detection images corresponding to the image above it. Subject numbers are indicated in the top right corner. All scale bars, 100 μm.

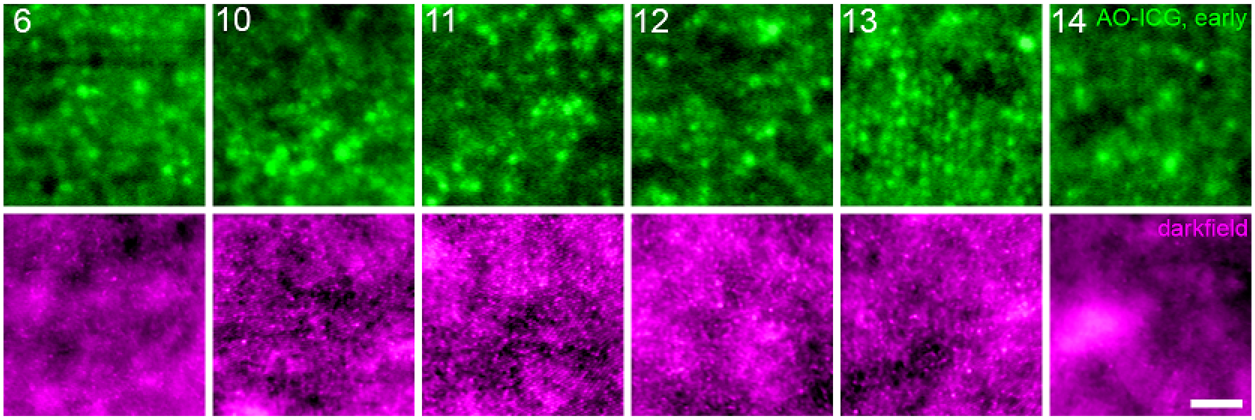

Supplementary Figure 2. RPE cells were better visualized using AO-ICG (top row) compared to darkfield (bottom row). Each column represents a different eye (subject number indicated in top left corner). In our hands, darkfield has a low success rate for visualizing RPE. Scale bar, 50 μm.

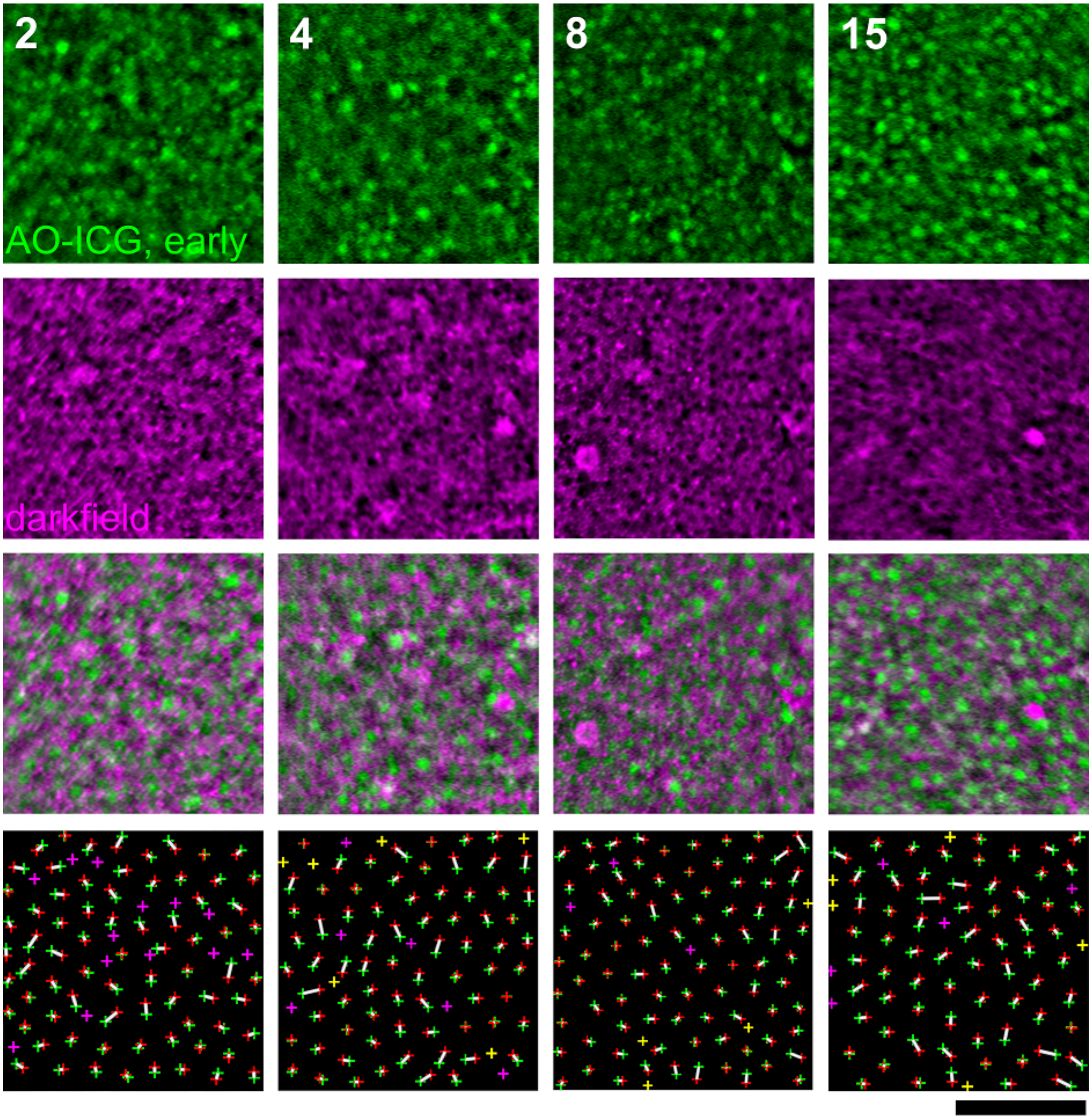

Supplementary Figure 3. Comparison of early-phase AO-ICG images of RPE cells (green) to darkfield images of RPE cells (magenta) in four subjects (codes indicated in top left corner). Images were enhanced as described in the Methods for visualization purposes. Color-merged images (green and magenta) confirm that the green AO-ICG signal is contained inside the magenta outlines of RPE cells. The bottom row shows the results of comparing AO-ICG images (green +s) to ground truth (red +s), with true correspondences between points indicated by connected white lines, false positives shown in yellow, and false negatives in magenta. Scale bar, 100 µm.

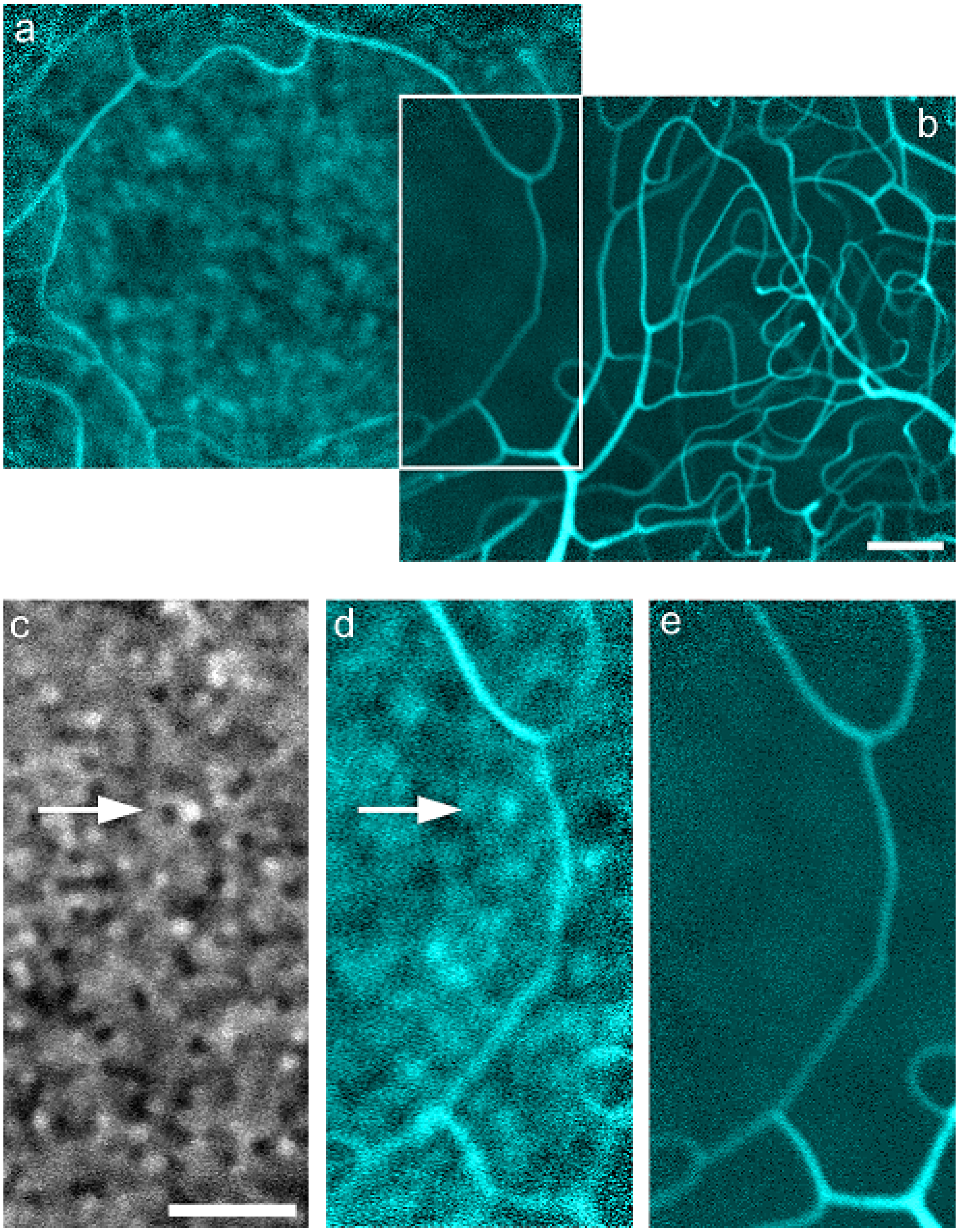

Supplementary Figure 4. Effect of optical sectioning on AO-ICGA images of inner retinal vessels (subject 5 from Supplementary Table 2). There was substantial out-of-focus fluorescent light originating from the underlying RPE in AO-ICG images acquired with (a) a large confocal aperture (weak rejection of out-of-focus light) compared to (b) a small confocal aperture (strong rejection of out-of-focus light). Images in (a) and (b) were taken 3 months apart with a repeat injection of ICG. (c-e) Zooms of overlapping area (white box) in (a) and (b). (c) Late phase AO-ICG image of RPE from visit 1. (d) There are some changes in the RPE pattern between the early (d) and late (c) phase, but the overall out-of-focus fluorescent pattern corresponding to the RPE can be seen (e.g. white arrow). (e) The out of focus light is significantly reduced using a smaller confocal aperture. This data also confirms that the signal that we are observing arises from the outer retina and is not the result of out of focus light from the inner retina. All scale bars, 100 µm.

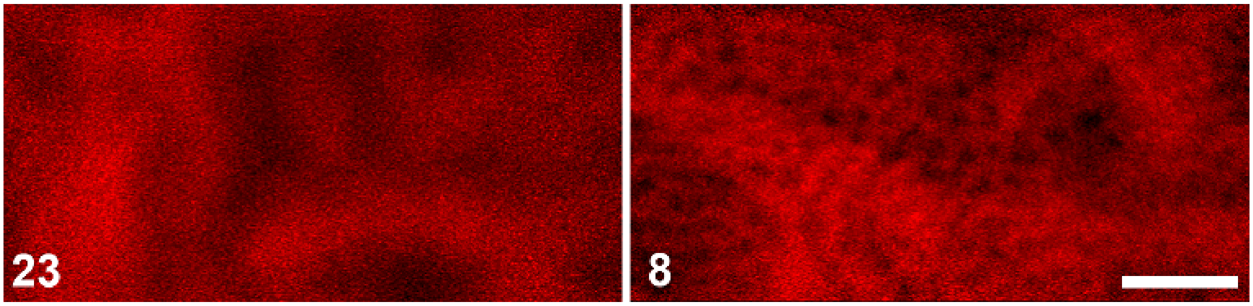

Supplementary Figure 5. Images of choroidal vessels at additional non-foveal locations (approximate retinal eccentricity: 4.5 mm temporal). The subject code is indicated in the bottom left corner. By changing the focus to a deeper layer beneath the choriocapillaris, it was possible to see larger choroidal vessels (subject 23). Out of focus light from the larger choroidal vessels can also been seen while focused at the choriocapillaris (subject 8). Scale bar, 100 μm.

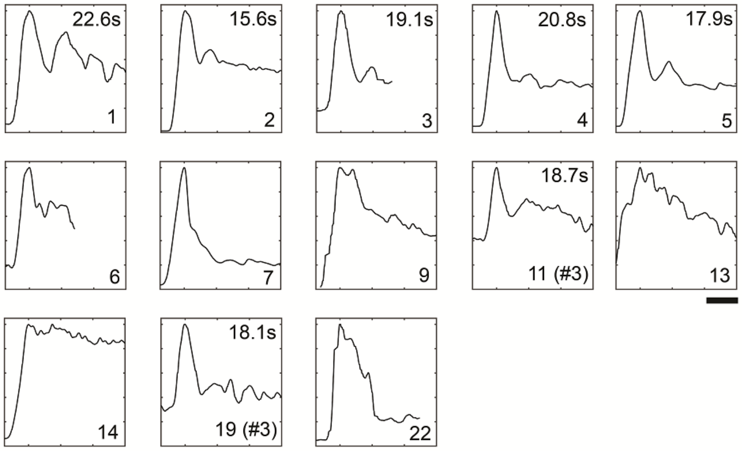

Supplementary Figure 6. AO-ICG fluorescence intensity time plot. Data from single push subjects as well as from the third push of three push (1+1+1) subjects are shown (subject number indicated in bottom right corner). The recirculation times measured are show in the top right corner. Some were not measurable as described in the text. Scale bar, 20 seconds.

Supplementary Table 1 – AO modalities

AO modality	Type of light	Imaging applications	Color
Confocal reflectance	Backscattered	Photoreceptors, RPE, nerve fibers, vasculature	Yellow
Non-confocal split detection[1]	Multiply-scattered	Photoreceptor inner segments, vasculature	Gray
Darkfield	Multiply-scattered	RPE	Magenta
AO-ICG, early phase[1]	Fluorescent	RPE	Green
		CC	Red
		Retinal vasculature	Cyan
AO-ICG, late phase	Fluorescent	RPE	Gray

1 To our knowledge, we show for the first time that this modality can also be used to visualize RPE cell structure.

Supplementary Table 2 - Subject Information

Subject[1]	Age	Sex	Eye	Dose[2]	Imaging Locations[3]	Data[4]
1	42	M	OS	3	Fovea	CC, FV, RT
2	24	F	OD	3	Fovea	CC, DF, FV, RT, PRC
3	33	M	OD	3	Fovea	RT, IRV
4	40	F	OD	3	Fovea	CC, DF, FV, RT, PRC
5	25	F	OS	3	Fovea	CC, FV, RT, IRV
6	47	F	OS	1+2	Fovea	CC, FV
7	27	M	OS	3	Fovea	CC, FV
8	43	F	OS	1+2	Fovea	CC, DF, FV, RT, PRC
9	21	F	OS	3	Fovea	CC, FV
10	24	F	OS	1+2	Fovea	CC, FV, RT
11	22	M	OD	1+1+1	Fovea	CC, FV, RT
12	28	F	OS	1+2	Fovea	CC, FV, RT
13	19	F	OD	3	Fovea	CC, FV
14	52	F	OS	3	Fovea	CC, FV
15	35	F	OS	1+2	Fovea, Parafovea	CC, DF, FV, RT, PRC
16	22	F	OS	1+2	Parafovea	RT, IRV
17	26	M	OS	1+2	Parafovea	RT, IRV
18	23	M	OD	1+2	Mid-periphery	RT
19	27	M	OS	1+1+1	Fovea	RT
20	39	F	OD	1+2	Fovea	RT
21	42	M	OD	1+2	Parafovea	RT
22	37	M	OD	3	Parafovea	CC
23	30	F	OS	1+2	Mid-periphery[5]	CC
RP	49	F	OS	1+2	Parafovea	CC, FV, RT, PRC

[1] # = subject with healthy eye, RP = patient with retinitis pigmentosa.

[2] ICG dose (25 mg in 3 mL), given in one push (3 mL), two pushes (1 mL followed by 2 mL), or three pushes (1 mL each).

[3] Fovea = preferred retinal locus of fixation.

[4] DF = darkfield comparison, CC = choriocapillaris visualization, FV = flow void segmentation, RT = recirculation time calculated, PRC = PR-RPE-CC quantification, IRV = inner retinal vasculature imaged

[5] Focus set to deeper than choriocapillaris

Supplementary Table 3 – Comparison of early-phase AO-ICG to darkfield images of RPE

Subject	n[1]	TP	FP	FN	Recall[2]	Precision[3]
2	87	74	0	13	0.85	1.00
4	79	72	7	7	0.91	0.91
8	89	86	4	3	0.97	0.96
15	73	67	6	6	0.92	0.92

[1] n = number of RPE cells identified from the darkfield image

[2] Recall = TP/(TP+FN)

[3] Precision = TP/(TP+FP)

Supplementary Table 4 – Quantification of foveal choriocapillaris flow voids

AO-ICG			
Subject	Area [μm^2]	Perimeter [µm]	Effective diameter [µm]
1	315 ± 118	58.3 ± 10.6	17.0 ± 3.4
2	312 ± 110	56.3 ± 11.0	16.8 ± 3.4
4	194 ± 48	43.4 ± 6.5	13.5 ± 2.2
5	310 ± 93	55.7 ± 9.3	16.8 ± 2.3
6	372 ± 115	62.9 ± 14.5	18.7 ± 3.3
7	385 ± 129	61.6 ± 10.5	19.0 ± 3.1
8	266 ± 69	52.1 ± 6.6	15.9 ± 1.9
9	281 ± 65	53.2 ± 6.7	16.2 ± 2.0
10	329 ± 98	57.6 ± 9.3	17.7 ± 2.5
11	300 ± 99	54.0 ± 10.0	16.7 ± 2.9
12	347 ± 118	58.9 ± 14.0	17.8 ± 2.7
13	322 ± 111	56.0 ± 10.0	17.4 ± 3.0
14	401 ± 142	62.8 ± 11.8	19.3 ± 3.3
15	245 ± 125	49.4 ± 13.5	14.7 ± 4.1
RP ROI 1[2]	288 ± 139	53.1 ± 14.6	15.9 ± 4.6
RP ROI 2	236 ± 123	49.5 ± 16.4	14.2 ± 4.4
Published histology[1]			
Subject	Area [μm^2]	Perimeter [µm]	Effective diameter [µm]
Olver [20]	127 ± 59	35.9 ± 11.6	9.8 ± 2.9
Yoneya [21]	234 ± 166	58.8 ± 35.0	14.3 ± 4.9
Zouache [22]	401 ± 391	67.7 ± 48.8	17.6 ± 7.4

[1] Macular human choriocapillaris only

[2] ROI 1: edge of preserved photoreceptors (eccentricity 0.75 mm); ROI2: in area of preserved RPE with no overlying photoreceptors (eccentricity 0.96 mm).

Supplementary Table 5. Power spectrum analysis of AO-ICG choriocapillaris images.

Subject	Row-to-row spacing [μm]
1	31.7
2	27.6
4	26.1
5	20.3
6	33.8
7	39.0
8	31.6
9	39.9
10	32.6
11	25.4
12	29.8
13	31.3
14	33.7
15	30.3

Supplementary Table 6. Measured size scales within the PR-RPE-CC complex.

Subject	PR[1] [μm]	RPE[2] [μm]	CC[3] [μm]
2	4.1	12.5	16.8
4	4.2	12.0	13.5
8	4.2	12.4	15.9
15	3.9	13.6	14.7
RP ROI 1	8.8	13.2	15.9
RP ROI 2	N/A	14.9	14.2

[1] Cell to cell spacing within a 37 μm x 37 μm ROI for healthy subjects and a 50 μm x 50 μm ROI for RP ROI 1.

[2] Cell to cell spacing within a 100 μm x 100 μm ROI.

[3] Effective diameter of flow voids within a 200 μm x 200 μm ROI.
