# Supplementary material for: Combining multimodal adaptive optics imaging and angiography improves visualization of human eyes with cellular-level resolution
Source: Commun Biol. 2018 Nov 14;1:189. doi: 10.1038/s42003-018-0190-8 (PMC6235967; doi:10.1038/s42003-018-0190-8)
Supplement: Supplementary file 3 — Description of additional supplementary items [file 42003_2018_190_MOESM3_ESM.docx]

**Description of Additional Supplementary Files**

**File Name**: Supplementary Data 1

**Description**: Source data for the plots shown in Figure 2C are provided in a supplementary Excel file.
